# Antifreeze Peptides Preparation from Tilapia Skin and Evaluation of Its Cryoprotective Effect on *Lacticaseibacillus rhamnosus*

**DOI:** 10.3390/foods11060857

**Published:** 2022-03-17

**Authors:** Yan Zeng, Weinan Li, Yu Liu, Wei Jiang

**Affiliations:** 1 Key Laboratory of Key Technical Factors in Zhejiang Seafood Health Hazards, Institute of Innovation & Application, Zhejiang Ocean University, Zhoushan 316022, China; 18434162948@163.com (Y.Z.); lwnzjou@163.com (W.L.); 2 Laboratory of Seafood Processing, Innovative and Application Institute, Zhejiang Ocean University, Zhoushan 316022, China; liuyu1987@zjou.edu.cn

**Keywords:** antifreeze peptide, *Lacticaseibacillus rhamnosus* ATCC7469, tilapia skin, cryoprotective mechanism

## Abstract

Antifreeze peptides can protect cell membranes and maintain the cell viability of probiotics under cold stress. Given this, antifreeze peptides were prepared from tilapia processing byproducts of tilapia skin by enzymolysis using the response surface methodology (RSM) method. The cryoprotective effects on *Lacticaseibacillus rhamnosus* ATCC7469 were investigated. Trypsin was selected as the protease for tilapia skin hydrolysis. The optimal hydrolysis conditions consisted of the amount of enzyme (2200 U/g), solid–liquid ratio (1:10, *w*/*v*), reaction temperature (49 °C), and reaction time (6.8 h), and the relative survival rate of *L. rhamnosus* reached 98.32%. Molecular weight (*M_w_*) distribution and peptide sequences of the antifreeze peptides prepared from tilapia skin (APT) under the optimal conditions were analyzed. APT significantly reduced the leakage of extracellular proteins and protected *β*-galactosidase and lactate dehydrogenase activities of *L. rhamnosus*. Compared with the saline group, scanning electron microscopy (SEM) observation showed that cells had a more normal, smooth, and entire surface under the protection of APT. These findings indicate that APT can be a new cryoprotectant in preserving probiotics.

## 1. Introduction

Probiotics are living microorganisms that can bring health benefits when given in proper amounts [1,2]. Nowadays, the most prevalent approach for using probiotics is the introduction of probiotics into the body employing food [3]. Compared with other probiotics, the most significant advantage of *L. rhamnosus* is that it produces only *L*-lactic acid, without other acids that may affect the quality of foods [4]. The benefits can be obtained when the viability of probiotics must maintain at more than 7 log CFU/mL in the foods [5,6]. However, the viability of probiotics may be affected by strain type, pH, and temperature [7]. Generally, the cytoplasmic membrane is of particular importance for the energy conversion systems of the cells. They depend on the state of lipid bilayers, which is influenced by changes in the external temperature. It was mentioned that the negative effect of cold stress was ascribed mainly by the physical action on the cell structures and enzymatic reactions [8]. Cells always experience a certain degree of damage during freezing and thawing processes, resulting in viability and metabolic activity loss. Morphological cell change and membrane damage may also be observed, causing leakage of nucleic acids and proteins [9].

Cryoprotectants are necessary to reduce mechanical damage during cryopreservation of probiotics [10]. Cryoprotectants are usually highly soluble in water and are relatively non-toxic to cells [11]. Nowadays, skim milk, sucrose, phosphates, glycerol, and dimethyl sulfoxide (DMSO) are widely and successfully used as antifreeze agents in the food industry [9]. However, some disadvantages of these antifreeze agents have attracted broad attention in research and industry areas. For instance, traditional antifreeze agents, such as skim milk, sucrose, and phosphates, may bring undesired taste and high energy, while glycerol and DMSO may cause irreversible toxic effects to probiotic cells [12,13]. Hence, innovative antifreeze agents which can overcome the above shortcomings should be investigated.

Antifreeze peptides are a category of protein hydrolysates that can protect frozen products under cold stress [10]. Antifreeze peptides show many typical properties, including thermal hysteresis ability [10], non-colligative reducing the freezing point [14], inhibiting crystal growth by binding to the surface of ice crystals [15], inhibiting recrystallization [16], and reducing cell damage [17]. These characteristics make antifreeze peptides have broad application prospects in frozen food systems [18]. Antifreeze peptides are reported in application such as frozen dough, frozen meat products, frozen fruit and vegetable products, dairy products, and cryobiology [10]. It has been reported that collagen hydrolysates from shark skin, pigskin, and tilapia scales were successfully used to protect lactic acid bacteria during the cold stage [15,19,20].

Tilapia is a vital aquaculture product in high-temperature areas and is commonly processed into frozen fillets [21]. Skin is an important part of byproducts during the processing of tilapia fillets, and it is mainly manufactured as animal feed at present. Since tilapia skin contains approximately 20% (*w*/*w*) protein, the preparation of functional peptides is a value-added way to utilize tilapia skin [22,23,24]. At present, antifreeze peptides prepared from tilapia skin have not been reported.

This study aimed to prepare antifreeze peptides from tilapia skin with high cryoprotective activity on *L. rhamnosus* during cryopreservation. Enzymatic hydrolysis conditions were optimized, and the hypothermia protection mechanism of the obtained antifreeze peptides was further analyzed.

## 2. Materials and Methods

### 2.1. Materials

Tilapia skin was provided by Hailisheng Group Co., Ltd. (Zhoushan, Zhejiang, China). *L. rhamnosus* ATCC7469 was provided by Shanghai Luwei Microbial Sci. & Tech. Co., Ltd. (Shanghai, China). MRS broth and MRS agar were obtained from Qingdao Hope Bio-Technology CO., Ltd. (Tsingtao, China). Dispase (50,000 U/g), alkaline proteinase (200,000 U/g), papain (100,000 U/g), compound proteinase (100,000 U/g), trypsin (250,000 U/g), BCA protein assay kit, *β*-GAL assay kit, and LDH assay kit were obtained from Beijing Solarbio Science & Technology Co., Ltd. (Beijing, China). The other reagents and chemicals were analytical grade.

### 2.2. Determination of Antifreeze Activity

The antifreeze activity determination refers to reported research with minor modifications [14,25]. *L. rhamnosus* activated overnight at 37 °C in MRS were cultured with an incubation dosage of 2% (*v*/*v*). Cells were obtained at the mid-log grown phase by centrifugation (8000 rpm and 4 °C for 10 min) and washed twice with saline. The cell density was diluted to OD_600_ of 1.0 by sterile saline. Diluted cells (0.1 mL) were transferred to a 2 mL centrifuge tube, where 0.9 mL of antifreeze peptides were added to achieve a final concentration of 1.0 mg/mL. The sterilized saline (0.9 mL) was used as a negative control. Positive controls included sucrose, skim milk, and glycerol with the final concentrations of 1 mg/mL, 1 mg/mL, and 20% (*v*/*v*), respectively. These centrifuge tubes were frozen at −20 °C for 24 h and thawed at 37 °C for 15 min. Then, 0.2 mL of each mixture was added in 3.8 mL of MRS and cultured at 37 °C for 20 h. *L. rhamnosus* relative survival rate was expressed as a percentage of cell concentration before and after freezing.

### 2.3. Screening of Proteases

The tilapia skin was thawed, washed, drained, and cut into pieces (1 cm × 1 cm). The prepared samples (5 g) were mixed with 0.2 mol/L NaOH (1:12, *w*/*v*) and shaken in a constant temperature shaker (120 r/min and 25 °C for 30 min). The treated skin was washed with water until pH 7.0. Then, the skin was immersed in 0.05 mol/L HCl (1:12, *w*/*v*), shaken in a constant temperature shaker (120 r/min and 25°C for 30 min) to swell the skins, and washed with water until pH 7.0 [26]. The treated tilapia skin was soaked in distilled water (1:12, *w*/*v*) and was adjusted to the designed pH values. The samples were hydrolyzed by dispase (pH 7.0, 55 °C), alkaline proteinase (pH 8.5, 50 °C), papain (pH 6.0, 50 °C), compound proteinase (pH 7.0, 50°C), and trypsin (pH 8.0, 40 °C) under optimum enzymolysis condition with 1500 U/g (enzyme activity-to-substrate) of enzyme for 6 h. Afterward, the enzymatic samples were heated to boiling for 15 min. The supernatants were collected and freeze-dried for further tests.

### 2.4. Optimization of Enzymatic Hydrolysis Conditions

Single-factor tests were conducted with the amount of enzyme (A, U/g), solid–liquid ratio (B, *w*/*v*), reaction temperature (C, °C), and reaction time (D, h). Optimal conditions for preparing antifreeze peptides from tilapia skins were carried out by RSM. The *L. rhamnosus* relative survival rate was chosen as the response index. The antifreeze peptides prepared under optimal conditions were named APT.

### 2.5. Determination of Molecular Weight (M_w_) Distribution

The *M_w_* distribution of APT was measured by HPLC (Agilent 1260, Santa Clara, CA, USA) with a TSKgel 2000SWXL column with the mobile phase (acetonitrile: water: TFA = 40:60:0.05, *v*/*v*/*v*), detected by the VWD detector at 220 nm.

### 2.6. Analysis of Amino Acid Composition

APT was digested with 6 mol/L HCl at 110 °C for 24 h under a nitrogen atmosphere. The obtained solution was dried under reduced pressure at 50 °C. The obtained sample was washed twice with distilled water. Finally, the dried samples were dissolved with sodium citrate buffer (pH 2.2) and filtrated through a 0.22 µm filter before the test. The amino acid composition was analyzed by an Amino Acid Analyzer (Hitachi L-8900, Tokyo, Japan).

### 2.7. Identification of Peptide Sequences

APT solutions were filtered through 3 kDa ultrafiltration centrifuge tubes (Millipore, MA, USA). After desalination, the components whose *M_w_* were under 3 kDa were subjected to sequence identification by Q Exactive HF-X (Thermo Scientific, Waltham, MA, USA) [27]. The peptide sequences were obtained by PEAKS Studio software (Bioinformatics Solutions Inc., Waterloo, ON, Canada).

### 2.8. Determination of β-GAL and LDH Activities

*L. rhamnosus* cell culture (5 mL) was centrifuged at 8000 rpm and 4 °C for 10 min and washed twice using saline. An equal volume of agents was added. Cells were frozen at −20 °C for 24 h. Thawed cells were then centrifuged at 8000 rpm and 4 °C for 10 min to discard the supernatant fractions and added 2 mL extract solution. The cells were destroyed through BILON-100 ultrasonic cell disruptor (Shanghai Bilon Instrument Co., Ltd., Shanghai, China) with the protection of an ice-bath. Then, the cell extract solutions were centrifuged at 4 °C and 10000 rpm for 10 min. The supernatants were used to measure *β*-GAL and LDH activities following the steps of the *β*-GAL assay kit and LDH assay kit provided by Beijing Solarbio Science & Technology Co., Ltd. (Beijing, China).

### 2.9. Assay of Extracellular Protein

*L. rhamnosus* cell suspension was prepared according to a previous method [9]. The cell density was diluted by sterile saline to OD_600_ of 1.2. Diluted cells (5 mL) were centrifuged to remove the saline. An equal volume of agents was added. The samples were frozen at −20 °C for 24 h. Two freeze–thaw cycles were performed every 2 h. After low-temperature treatment, the supernatant fractions were collected by centrifuging (8000 rpm and 4 °C for 10 min). The contents of extracellular protein were analyzed with the BCA protein assay kit [28].

### 2.10. Scanning Electron Microscopy

The cells washed twice in sterile saline were frozen at −20 °C for 24 h with saline or APT and then freeze-dried. The freeze-dried cells were covered with a small amount of gold and observed in a Zeiss Sigma 300 scanning electron microscope (Carl Zeiss, Oberkochen, Germany).

### 2.11. Statistical Analysis

Statistical analyses were performed using the IBM SPSS Statistics 26 software (SPSS Inc., Chicago, IL, USA). Significance analysis was performed by ANOVA with the Tukey test. Design Expert 8.0.6 (Stat-Ease Inc., Minneapolis, MN, USA) software was used for RSM analysis. All the measurements were carried out in triplicate.

## 3. Results and Discussion

### 3.1. Selection of Protease

**Figure 1:**
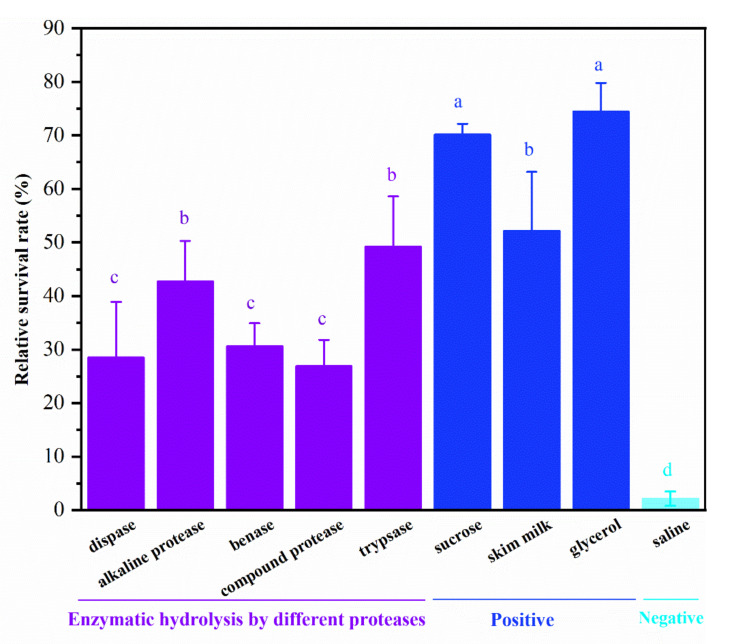
The antifreeze activities of different enzymatic hydrolysis samples (1 mg/mL), positive controls (1 mg/mL sucrose, 1 mg/mL skim milk, and 20% (*v*/*v*) glycerol), and negative control (sterilized saline) on *L. rhamnosus* frozen at −20 °C for 24 h. The bars with different letters indicate significant differences (*p* < 0.05).

The tilapia skins were treated by five commercial proteases, resulting in different enzymatic hydrolysis products. Antifreeze protective activities on *L. rhamnosus* of five freeze-dried enzymatic hydrolysis samples, three positive control groups, and one negative control group are shown in Figure 1. The positive control 20% glycerol showed the highest relative survival rate of 74.49%, followed by the positive control 1 mg/mL sucrose with the relative survival rate of 70.20%. However, the negative control sterilized saline exhibited the lowest relative survival rate of 2.14%. The results indicate that antifreeze protectant may be an excellent tool to protect probiotics during freezing. Among five freeze-dried enzymatic hydrolysis samples, the trypsin hydrolysate and alkaline protease showed significantly higher cryopreservation activity (with relative survival rates of 49.24% and 42.72%, respectively) than the other protease hydrolysates (*p* < 0.05). Besides, the trypsin hydrolysate exhibited a similar antifreeze protective activity to the positive control 1 mg/mL skim milk (with relative survival rates of 52.17%). Antifreeze peptides prepared from tilapia scales by trypsin hydrolysis of collagen could alleviate the metabolic activity reduction in *Streptococcus thermophilus* during frozen storage [9]. The specificity of trypsin determines the action sites of its catalytic hydrolysis, which may lead to higher antifreeze activity of enzymatic hydrolysis substrate with trypsin than other enzymes. Thus, trypsin was selected as the optimal protease for tilapia skin hydrolysis in the subsequent research.

### 3.2. Optimization of Enzymatic Hydrolysis Conditions

#### 3.2.1. Single-Factor Experiments

The single-factor experiments were carried out to obtain the proper ranges of the amount of enzyme (A, U/g), solid–liquid ratio (B, *w*/*v*), reaction temperature (C, °C), and reaction time (D, h). Initially, the effect of amounts of enzyme (500–2500 U/g) was studied with solid–liquid ratio (1:12, *w*/*v*), reaction temperature (50 °C), and reaction time (6 h). The results showed that the highest relative survival rate of *L. rhamnosus* was obtained when the amount of enzyme increased up to 2000 U/g, and the relative survival rate went down as the amount of enzyme further increased. The second step was to determine the effect of solid–liquid ratio (1:8–1:16, *w*/*v*) with the fixed amount of enzyme (2000 U/g) obtained from the first step, reaction temperature (50 °C), and reaction time (6 h). The highest relative survival rate was achieved at 1:10. The next step was to determine the effect of reaction temperatures (40–60 °C) with solid–liquid ratio (1:10, *w*/*v*), amount of enzyme (2000 U/g), and reaction time (6 h). The highest relative survival rate was achieved at 50 °C. Finally, the optimum reaction time (4–8 h) at the fixed amount of enzyme (2000 U/g), solid–liquid ratio (1:10), reaction temperature (50 °C) was determined to be 6 h. Based on the single factor tests, the four variables and three levels of tests were designed in Table S1.

#### 3.2.2. Establishing the Fitted Model

AS shown in Table S2, the software designed a total of 29 groups of enzymolysis conditions. The relative survival rate of *L. rhamnosus* was used as the response index. The following polynomial stepwise equation was obtained by multiple regression analysis:

Y = 92.93 + 5.68A + 0.46B + 1.26C + 2.83D − 1.78AB − 2.48AC − 1.23AD − 3.13BC + 0.84BD − 1.05CD − 5.62A^2^ − 3.77B^2^ − 2.97C^2^ − 1.59D^2^ (1)

where Y is the relative survival rate of *L. rhamnosus*, A is the amount of enzyme, B is the solid–liquid ratio, C is the reaction temperature, and D is the reaction time.

As shown in Table 1, the linear variable A was statistically significant (*p* < 0.01). The linear variables D, and the quadratic variables A^2^ and B^2^, were also statistically significant (*p* < 0.05). The influence of each factor on the relative survival rate of *L. rhamnosus* was ordered as A > D > C > B. The model was statistically significant (*p* = 0.0048 < 0.05), and lack of fit was not significant (*p* = 0.1016 > 0.05). Therefore, the proposed model adequately represents the connections between the independent variables (amount of enzyme, solid–liquid ratio, reaction temperature, and reaction time) and the response index (relative survival rate of *L. rhamnosus*).

#### 3.2.3. Response Surface Analysis

**Figure 2:**
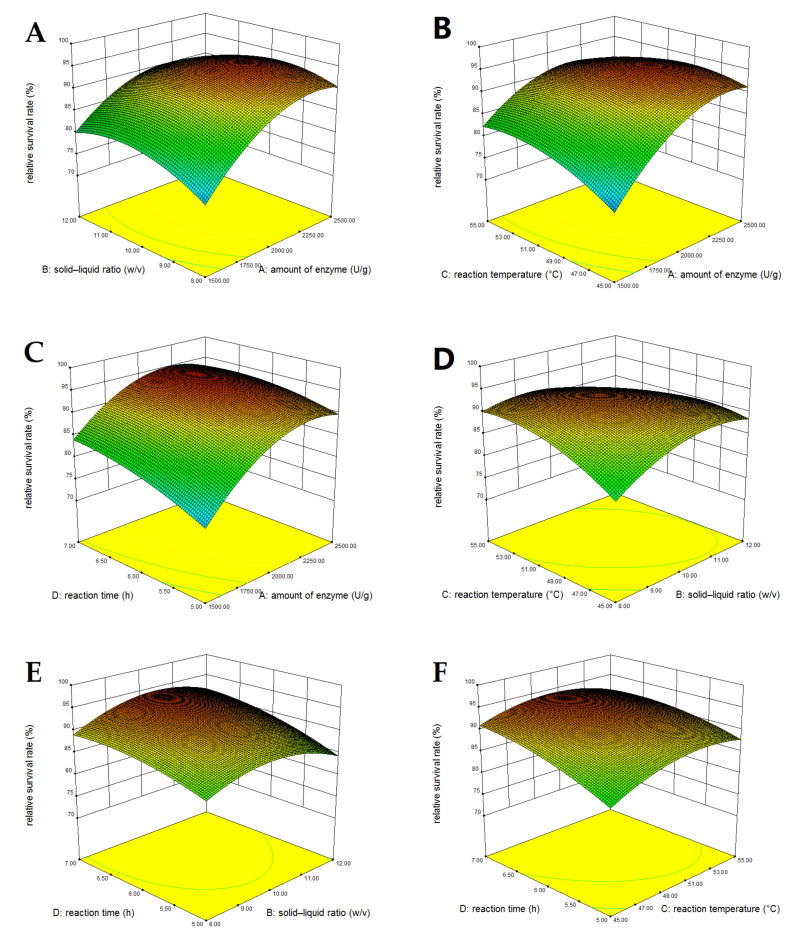
The interaction of independent variables on the relative survival rate of *L. rhamnosus* frozen at −20 °C for 24 h. The interaction between two independent variables were as follows: (**A**), the solid–liquid ratio and amount of enzyme; (**B**), the reaction temperature and amount of enzyme; (**C**), the reaction time and amount of enzyme; (**D**), the reaction temperature and solid–liquid ratio; (**E**), the reaction time and solid–liquid ratio; (**F**), the reaction temperature and reaction time.

Three-dimensional response surface plots might be helpful to illustrate the primary and interactive effects of independent variables on the response index. Figure 2A−F displays the influence of two independent variables on the relative survival rate while the remaining two were kept constant at their zero levels. Figure 2A−C shows the effects of the amount of enzyme along with any of the other three factors on the relative survival rate. The interactions between the amount of enzyme and the other three factors were relatively significant, as reflected by the steep surfaces. Other interactions on the relative survival rate were not meaningful, as reflected by the flat surfaces.

#### 3.2.4. Optimal Conditions and Verification

The theoretical optimal conditions for hydrolysis were obtained as follows: the amount of enzyme (2218.45 U/g), solid–liquid ratio(1:10.25, *w*/*v*), reaction temperature (49.1 °C), and reaction time (6.81 h). The predicted relative survival rate of *L. rhamnosus* was 95.24% in these conditions. The experimental verification conditions were set as 2200 U/g, 1:10, 49 °C, and 6.8 h, respectively. The relative standard deviation between the predicted relative survival rate (95.24%) and experimental value (98.32 ± 1.79%) was less than 5%. Therefore, the optimal conditions for antifreeze peptides preparation from tilapia skin were as follows: the amount of enzyme (2200 U/g), solid–liquid ratio (1:10, *w*/*v*), reaction temperature (49 °C), and reaction time (6.8 h). The relative survival rate of *L. rhamnosus* of the optimized antifreeze peptides (98.32%) was much higher than that of the unoptimized antifreeze peptides in Figure 1 (74.49%).

### 3.3. M_w_ Distribution and Amino Acid Composition of APT

The freeze-dried enzymatic hydrolysis samples produced under the optimum enzymolysis conditions were named APT. The standards used for the relative molecular weight correction curve include cytochrome C (*M_w_* 12355 Da), aprotinin (*M_w_* 6511 Da), bacitracin (*M_w_* 1422 Da), Gly-Gly-Tyr-Arg (*M_w_* 451 Da), and Gly-Gly-Gly (*M_w_* 189 Da). To investigate the *M_w_* distribution of APT, the logarithm of *M_w_* (lg *M_w_*) was plotted against the retention time (*R_t_*): lg *M_w_* = −0.2302 *R_t_* + 6.7432.

**Figure 3:**
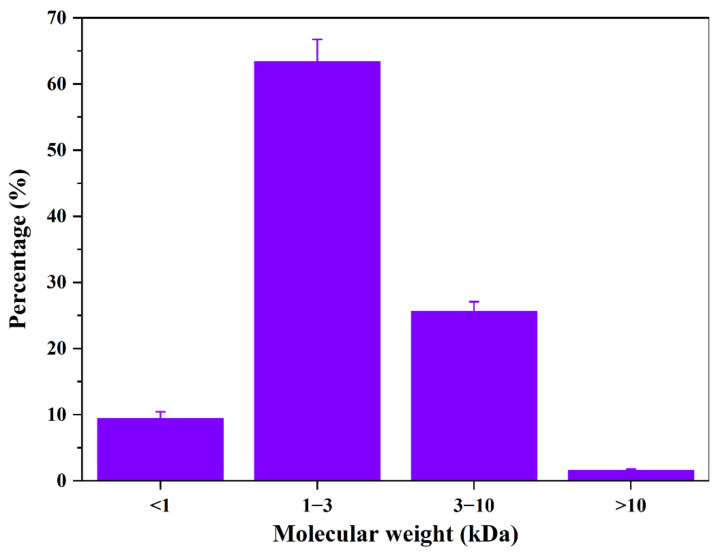
The molecular weight distribution of APT.

The measuring coefficient (*R*^2^) of the calibration equation was 0.9658, indicating an acceptable fitting relationship between the compounds with *M_w_* ranging from 189 Da to 12355 Da. Figure 3 shows the molecular weight distribution of APT, indicating that the *M_w_* fractions mainly were distributed between 1 kDa and 3 kDa (accounted for 63.37%). The *M_w_* of 3 k–10 kDa accounted for 25.64%. Wu et al. previously isolated antifreeze peptides from sericin with the *M_w_* of 1009.50 Da [29]. Meanwhile, Wang et al. obtained antifreeze peptides from pigskin with the *M_w_* distribution of 150–2000 Da [15]. The *M_w_* distribution of protein hydrolysates determines their biological and functional properties [30]. Antifreeze peptides with a certain molecular dimension might display significant cryoprotective activity by inhibiting recrystallization after absorbing to ice surfaces [16]. Furthermore, peptides with *M_w_* lower than 2000 Da can maintain the integrity of the cells and protect the cells from cold stress [31]. The results agreed with previous research that short peptides can adsorb to the ice and inhibit crystallization [32].

The amino acid composition of APT is given in Table 2. APT was rich in Gly (35.45%), Ala (13.29%), and Pro (12.43%). It was reported that the antifreeze activity of proteins was related to the contents of Gly and Ala [33,34]. Besides, the alkyl side chains of Pro and Ala residues in antifreeze peptides provided a part of the non-polar environment. They maintained the hydrogen bonds formed by antifreeze peptides’ hydrophobic interaction with water molecules, resulting in an inhibiting effect on ice crystal growth [10]. The amino acid composition of APT was similar to the antifreeze peptides prepared from the *Scomberomorus niphonius* skin [35]. The results indicate that Gly, Pro, and Ala may responsible for the antifreeze activity of APT.

### 3.4. Peptide Sequences of APT

In general, *M_w_*, amino acid composition, and peptide sequence are the key structural properties that affect the antifreeze activity of peptides [36,37]. To explore which peptides exhibited antifreeze activity, we identified the peptide sequences of APT. Table 3 shows the top 20 abundance identified peptide sequences of APT, whose *M_w_* were in the range of 632–2939 Da. It was reported that short peptides (600–2700 Da) were easily adsorbed to the ice surface and inhibited recrystallization [19]. Thus, 20 peptides might play a critical role in protecting *L. rhamnosus* during freezing. Based on the NCBI database, the 20 peptides mentioned above were from various collagen, lumican, and decorin. Previous research showed that antifreeze glycopeptide analogs obtained by nonenzymatic glycation could improve the antifreeze activity [14]. Lumican and decorin are both small leucine-rich repeat proteoglycans [38]. Leucine is a hydrophobic amino acid, so these peptides derived from glycoprotein might inhibit ice crystal growth by their hydrophobic action. Antifreeze peptides commonly have particular sequence characteristics, including a tripeptide repeat sequence (-Gly-X-Y-). The result of sequence analysis indicated that many identified peptides were consistent with structural characteristics. Therefore, some of the peptides in Table 3 might be regarded as cryoprotectants. Nevertheless, additional research is necessary to analyze the antifreeze activity and the cryoprotective effect on *L. rhamnosus* through the purification or synthesis of sufficient quantities of these peptides.

### 3.5. Effect of APT on Cell Metabolic Activity

**Figure 4:**
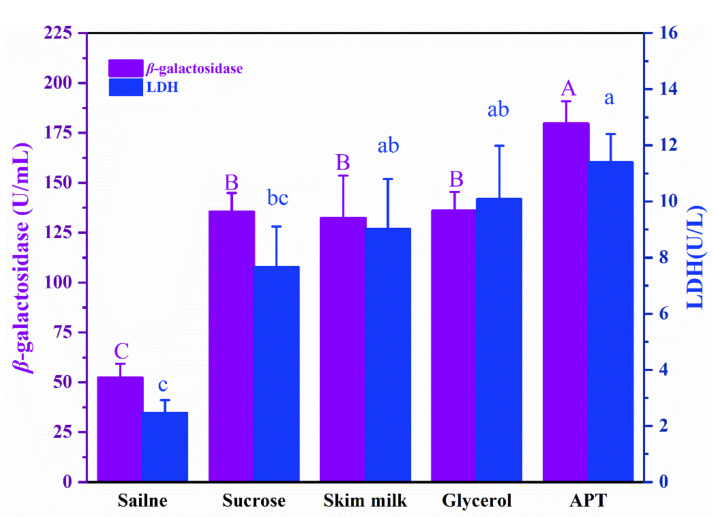
Effects of saline, positive cryoprotectants, and APT on *β*-galactosidase (*β*-GAL) and lactic dehydrogenase (LDH) activities of *L. rhamnosus* frozen at −20 °C for 24 h. The bars in the same color with different capital letters or lowercase letters indicate significant differences (*p* < 0.05).

*β*-galactosidase (*β*-GAL) transforms glucose and galactose into lactose, which is important for the probiotic effects of lactic acid bacteria (LAB) [39]. Lactic dehydrogenase (LDH) is critical to LAB metabolism, catalyzing pyruvate to lactic acid. Effects of saline, positive cryoprotectants, and APT on the activities of *β*-GAL and LDH were investigated, and the results are shown in Figure 4. With the protection of APT, the activities of *β*-GAL and LDH (179.79 U/mL and 11.41 U/L, respectively) were significantly higher than the saline group (52.43 U/mL and 2.48 U/L, respectively) and three positive cryoprotectants (132.47–135.60 U/mL and 7.68–10.09 U/L, respectively) (*p* < 0.05). The results indicate that APT is an effective cryoprotectant for maintaining the cell metabolic activity of freezing *L. rhamnosus*. The protective effect of AKAP on *β*-GAL and LDH activities might be attributed to the maintenance of cell membranes and prevent formation of the intracellular ice, which was similar to the antifreeze mechanism of disaccharides [40].

### 3.6. Effect of APT on Cell Membrane Permeability

**Figure 5:**
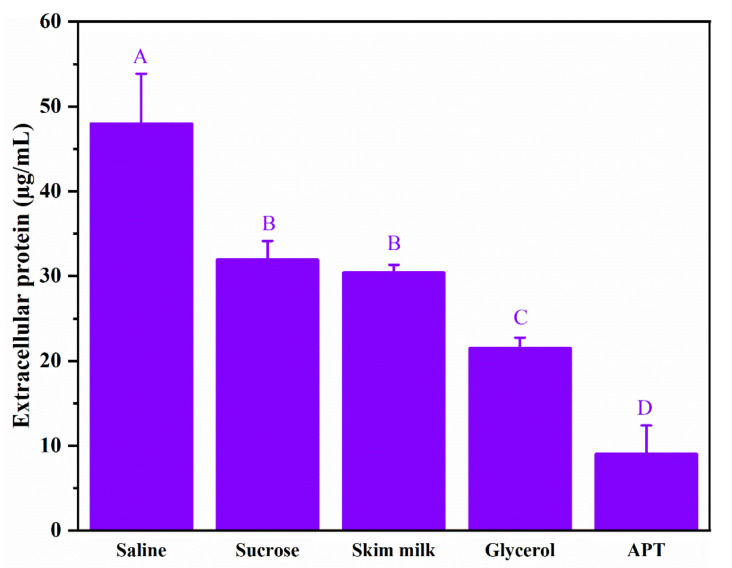
Effects of saline, positive cryoprotectants, and APT on the proteins leakage of *L. rhamnosus* after frozen at −20 °C for 24 h. The bars with different lowercase letters indicate significant differences (*p* < 0.05).

**Figure 6:**
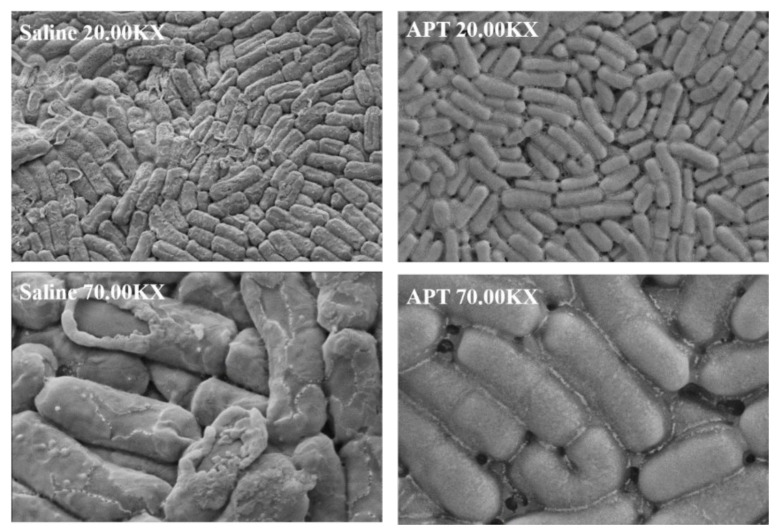
The scanning electron micrographs of *L. rhamnosus* frozen at −20 °C for 24 h with saline (**left**) and APT (**right**).

Ice crystals’ growth during freezing may cause mechanical damage and cell membrane rupture, leading to membrane damage and leakage of intracellular proteins. The results of extracellular proteins of *L. rhamnosus* after being frozen at −20 °C for 24 h are shown in Figure 5. During freeze–thawing steps, the extracellular protein content in the saline group reached 48.01 µg/mL, which was significantly higher than the three positive cryoprotectants and APT (*p* < 0.05). It suggested that freezing destroyed the cellular membrane and led to leakage of intracellular proteins without adding a cryoprotectant. The lowest extracellular protein content was observed in the APT group (9.02 µg/mL), which was significantly lower than the three positive cryoprotectants and saline (*p* < 0.05). As shown in Figure 6, the surface morphology of *L. rhamnosus* frozen with saline exhibited severe ruptures and deformation, enhancing membrane permeability. In contrast, the *L. rhamnosus* cells with APT protection were covered in a thin layer and exhibited a normal, smooth, and round appearance. The scanning electron microscopy results verified the phenomenon of intracellular protein leakage in Figure 5. To sum up, the results indicate that APT protected the integrity of the cell membrane and alleviated the intracellular protein leakage in freezing *L. rhamnosus*.

## 4. Conclusions

The enzymatic hydrolysis condition of APT was optimized by RSM, and the relative survival rate of *L. rhamnosus* was increased from 74.49% to 98.32 %. With the protection of APT, *L. rhamnosus* cells had a more normal, smooth, and round surface compared with the saline groups. APT could protect the activities of *β*-GAL and LDH, and significantly reduce the leakage of extracellular proteins (*p* < 0.05). These findings indicate that APT likely effectively maintained the structural integrity of the cell membrane and protected cell viability. Based on the results, APT may be a new cryoprotectant for *L. rhamnosus* ATCC7469 or other probiotics. Furthermore, the application of APT in different food systems will be investigated in future research.

## Figures and Tables

**Table 1:** Analysis of variance (ANOVA) for the quadratic polynomial model.

Source	Sum of Squares	df	Mean Square	*F* Value	*p*-Value Prob > F	Significant
Model	863.9864	14	61.71332	4.32713	0.0048	*
A	387.717	1	387.717	27.18541	0.0001	**
B	2.585408	1	2.585408	0.18128	0.6767	
C	18.97567	1	18.97567	1.33051	0.2680	
D	95.93707	1	95.93707	6.726785	0.0212	*
AB	12.70922	1	12.70922	0.891128	0.3612	
AC	24.6016	1	24.6016	1.724981	0.2102	
AD	6.0516	1	6.0516	0.424318	0.5253	
BC	39.12502	1	39.12502	2.743315	0.1199	
BD	2.839225	1	2.839225	0.199077	0.6623	
CD	4.41	1	4.41	0.309214	0.5869	
A^2^	204.7503	1	204.7503	14.3564	0.0020	*
B^2^	92.11029	1	92.11029	6.458463	0.0235	*
C^2^	57.05622	1	57.05622	4.00059	0.0653	
D^2^	16.36413	1	16.36413	1.147397	0.3022	
Residual	199.6673	14	14.26195			
Lack of Fit	181.0135	10	18.10135	3.881537	0.1016	not significant
Pure Error	18.6538	4	4.66345			
Cor Total	1063.654	28				

* The model or variable is significant (*p* < 0.05); ** The variable is significant (*p* < 0.01).

**Table 2:** Amino acid composition of APT.

Amino Acid	Molar Percentage (%)	Amino Acid	Molar Percentage (%)
Asp	5.12	Met	1.08
Thr	2.83	Ile	1.11
Ser	3.94	Leu	2.69
Glu	8.64	Tyr	0.50
Pro	12.43	Phe	1.50
Gly	35.45	Lys	2.76
Ala	13.29	His	0.62
Cys	0.05	Arg	5.80
Val	2.20		

**Table 3:** List of the peptide sequences identified from APT and the associated proteins of the peptides.

NO.	Peptide	Length	Mass	m/z	−10lgP	Protein Name
1	GPTGEIGATGLAGAR	15	1326.689	443.2371	45.47	collagen alpha-2(I) chain precursor
2	RGPTGEIGATGLAGAR	16	1482.79	371.7053	49.78	collagen alpha-2(I) chain precursor
3	GLSGNIGFPGPK	12	1158.603	580.3095	24.14	collagen alpha-2(I) chain precursor
4	SPAMPVPGPMGPMGPR	16	1577.752	526.9243	52.54	collagen alpha-1(I) chain precursor
5	WLIIDNNQITNAK	13	1541.82	514.9476	43.46	lumican
6	SSGPPVPGPIGPMGPR	16	1501.771	501.5981	52.57	collagen, type I, alpha 1b precursor
7	GLQGFVGLPGSR	12	1202.641	602.3278	26.16	collagen alpha-2(I) chain precursor
8	LVDSGIPAGVF	11	1073.576	1074.583	32.6	lumican
9	GPPGPMGPPGLAGAPGEPGR	20	1783.867	892.9423	56.6	collagen alpha-1(I) chain precursor
10	FMGPLNY	7	840.384	841.3916	35.48	lumican
11	GLTGPIGLPGPAGSPGDKGEPGAQGPVGPSGAR	33	2939.474	1470.744	58.95	collagen alpha-1(I) chain precursor
12	GPTGEIGATGL	11	971.4924	972.4996	39.71	collagen alpha-2(I) chain precursor
13	RGPTGEIGATGL	12	1127.594	376.8716	30.39	collagen alpha-2(I) chain precursor
14	WEVQPVTF	8	1004.497	1005.504	36.73	decorin precursor
15	FPSGLL	6	632.3533	633.361	22.69	lumican
16	GFPGLAGPVGEPGK	14	1297.667	649.8418	38.04	collagen alpha-1(I) chain precursor
17	PGPGPMGLM	9	855.3983	856.4062	36.06	collagen alpha-2(I) chain precursor
18	SPAMPVPGPM	10	982.4616	983.4692	46.85	collagen alpha-1(I) chain precursor
19	GDNGPPGLTGFPGAAGR	17	1571.733	786.8741	21.81	collagen alpha-2(I) chain precursor
20	MVQPQEKAPDPFR	13	1540.734	771.3766	30.45	collagen alpha-1(I) chain precursor

## Data Availability

Not applicable.

## Supplementary Materials

The following supporting information can be downloaded at: https://www.mdpi.com/article/10.3390/foods11060857/s1, Table S1: Enzymatic hydrolysis variable and levels for central composite design; Table S2: Experimental design and results.
