# Supplementary material for: Antifreeze Peptides Preparation from Tilapia Skin and Evaluation of Its Cryoprotective Effect on Lacticaseibacillus rhamnosus
Source: Foods. 2022 Mar 17;11(6):857. doi: 10.3390/foods11060857 (PMC8953377; doi:10.3390/foods11060857)
Supplement: Supplementary file 1 [file foods-11-00857-s001.zip › foods-1631305-supplementary.pdf]

Table S1. Enzymatic hydrolysis variable and levels for central composite design

Level	Amount of enzyme (A, U/g)	Solid-liquid Ratio (B, w/v)	Temperature (C, °C)	Time (D, h)
-1	1500	1:8	45	5
0	2000	1:10	50	6
1	2500	1:12	55	7

Table S2. Experimental design and results

No.	Amount of enzyme (A, U/g)	Solid-liquid Ratio (B, w/v)	Temperature (C, °C)	Time (D, h)	Relative survival rate（%）
1	2000	1:8	50	7	89.33
2	2000	1:8	50	5	87.69
3	2500	1:8	50	6	90.33
4	2000	1:8	45	6	82.13
5	1500	1:10	45	6	79.24
6	2000	1:10	50	6	93.53
7	2000	1:12	50	5	88.41
8	2500	1:10	50	7	92.57
9	2000	1:12	55	6	84.26
10	2000	1:10	50	6	93.15
11	2000	1:8	55	6	91.49
12	2000	1:10	45	7	92.15
13	1500	1:10	50	5	76.67
14	2000	1:12	50	7	93.42
15	1500	1:12	50	6	75.79
16	2000	1:10	55	5	82.17
17	2000	1:10	45	5	79.63
18	2500	1:10	50	5	91.81
19	2000	1:10	55	7	90.49
20	2500	1:10	45	6	89.73
21	2000	1:10	50	6	95.80
22	2500	1:12	50	6	88.12
23	1500	1:10	50	7	82.35
24	2500	1:10	55	6	88.77
25	2000	1:10	50	6	92.35
26	1500	1:8	50	6	70.87
27	2000	1:12	45	6	87.41
28	1500	1:10	55	6	88.20
29	2000	1:10	50	6	89.82
